# Analysis of Preoperative Detection for Apex Prostate Cancer by Transrectal Biopsy

**DOI:** 10.1155/2013/705865

**Published:** 2013-02-21

**Authors:** Tomokazu Sazuka, Takashi Imamoto, Takeshi Namekawa, Takanobu Utsumi, Mitsuru Yanagisawa, Koji Kawamura, Naoto Kamiya, Hiroyoshi Suzuki, Takeshi Ueda, Satoshi Ota, Yukio Nakatani, Tomohiko Ichikawa

**Affiliations:** ^1^Department of Urology, Graduate School of Medicine, Chiba University, 1-8-1 Inohana, Chuou-ku, Chiba 260-8670, Japan; ^2^Division of Urology, Chiba Cancer Center, Chiba 260-8717, Japan; ^3^Department of Urology, Toho University Sakura Medical Center, Sakura 285-8741, Japan; ^4^Department of Pathology, Graduate School of Medicine, Chiba University, Chiba 260-8670, Japan

## Abstract

*Background*. The aim of this study was to determine concordance rates for prostatectomy specimens and transrectal needle biopsy samples in various areas of the prostate in order to assess diagnostic accuracy of the transrectal biopsy approach, especially for presurgical detection of cancer in the prostatic apex. *Materials and Methods*. From 2006 to 2011, 158 patients whose radical prostatectomy specimens had been evaluated were retrospectively enrolled in this study. Concordance rates for histopathology results of prostatectomy specimens and needle biopsy samples were evaluated in 8 prostatic sections (apex, middle, base, and transitional zones bilaterally) from 73 patients diagnosed at this institution, besides factors for detecting apex cancer in total 118 true positive and false negative apex cancers. *Results*. Prostate cancer was found most frequently (85%) in the apex of all patients. Of 584 histopathology sections, 153 (49%) from all areas were false negatives, as were 45% of apex biopsy samples. No readily available preoperative factors for detecting apex cancer were identified. *Conclusions*. In Japanese patients, the most frequent location of prostate cancer is in the apex. There is a high false negative rate for transrectal biopsy samples. To improve the detection rate, transperitoneal biopsy or more accurate imaging technology is needed.

## 1. Introduction

One of the most frequent location of cancer in the prostate gland is in the apex. Iremashvili et al. showed the incidence of carcinoma in prostatectomy specimens; 65.4% of all patients had apex carcinoma, 56.6% had middle carcinoma, 47.3% had base carcinoma [1]. Apex core specimens obtained by needle biopsy have been associated with the highest cancer detection rates [2]. However, to the best of our knowledge, there have been no previous reports of assessments of the sensitivity and specificity of transrectal biopsy procedures for detection of apical prostate cancer through determining correlations between histopathologic diagnoses of preoperative transrectal biopsy and subsequently resected tissue specimens, especially with regard to presurgical detection of prostate cancer localized to the apex.

Recently, in Japan, prostate cancer (PCA) screening has spread and diagnostic imaging technology has improved. Detection of early stage PCA has been increasing [3, 4]. Kikuchi et al. reported that, in the United States after 1995, many smaller PCAs detected were located in the apex of the prostate: the frequency of apical cancer detection after 1995 had risen to 46% from 26%, a significant increase [5, 6]. Takashima et al. in 2002 reported that in Japanese men, 82.3% of all T1c prostate tumors were located in the apex and were significantly denser compared to midprostate tumors [7]. Because of such recent diagnostically related data, determination of precise tumor location is now a useful tool for patient care.

The protocol for systematic transrectal biopsy was introduced by Hodge et al. more than 20 years ago [8]; use of this technique has increased the PCA detection rate. Huo et al. reported that accuracy of biopsy core analysis, when correlated with prostatectomy specimens, had an average sensitivity and specificity for location of 48% and 84%, respectively [9], and Rogatsch et al. found a positive predictive value of only 71.1% [10]. Thus, predicting location by core specimen analysis has not been particularly reliable. 

Here we report results of a study of 14-core transrectal prostate biopsy specimens, 3 peripheral zone at regular intervals X 2 and 1 TZ X 1-X 2 bilaterally. The location of each cancer was determined from examination of subsequent radical prostatectomy (RP) specimens, and then concordance rates for prostatectomy specimens and preoperative needle biopsy samples of 8 prostate areas (bilateral apex, middle, base, and TZ) were determined, with special attention paid to detection of apex cancers by transrectal apex biopsy. 

## 2. Materials and Methods

A total of 158 patients whose RP specimens had been evaluated appropriately in 203 underwent RP patients at Chiba University Graduate School of Medicine, Japan, from 2006 to 2011 were retrospectively enrolled in this study. The study was performed with approval of the hospital ethics committee, and informed consent was obtained from patients. All patients had increased prostate specific antigen (PSA) levels (3.0 ng/mL or greater) and/or abnormal digital rectal examination (DRE) findings, and PCA diagnosed by needle biopsy. Patients who received neoadjuvant androgen deprivation therapy were excluded.

The indication for RP was clinically localized prostate cancer in patients aged 75 years or younger. Clinical stage T3 was also considered an indication for surgery. The clinicians considered not only clinical stage but also the Gleason score and PSA level.

Initial histopathology results were reported by experienced uropathologists after assessment of each prostate specimen, all of which were fully embedded and sectioned at 5 mm intervals for analysis. The anatomical locations of tumor foci were reproduced on a prostate cancer map. Tumor volumes were calculated using Image Processing and Analysis in JAVA (Image J, NIH, United States). We defined the prostatic apex tumor as all or a part of tumor located within 1 cm from distal end of radical prostatectomy specimen.

Transrectal ultrasound (TRUS) was performed using the SSD-2000 System and a 7.5-MHz transducer (Aloka, Japan). All patients received a local anesthesia injection (5 mL 1% lidocaine) to the apex of the prostate. Prostate needle biopsies were performed transrectally using an 18-gauge biopsy needle and a biopsy gun under TRUS guidance, providing 17 mm long tissue cores. For the 14-core biopsy, 12 specimens were taken from the peripheral zone at regular intervals and 2 specimens were taken from the TZs. All biopsy specimens were labeled according to the biopsy site (apex, middle, or base of the peripheral zone or TZ, and left or right lobe) and were then submitted in separate formalin-filled containers to the Department of Pathology, Chiba University Hospital.

The location of each cancer was determined in all cases, and concordance rates for prostatectomy specimens and needle biopsy samples from 8 sections (bilateral apex, middle, base, and TZ) were determined for 73 patients diagnosed at our institution. Clinicopathological factors possibly correlating with detection of apex cancer using transrectal biopsy were assessed in total 118 cancers, 65 true positive and 53 false negative apex cancers. 

Statistical analysis was performed using the Student's *t*-test, *χ* ^2^ test, Mann-Whitney *U* test, and logistic regression analysis. *P* values <0.05 were considered significant. SPSS version 12.0 software (SPSS, Chicago, Illinois, USA) was used for all analyses.

## 3. Results

All 158 consecutive patients receiving RP were included in this study. Clinical and pathological features are summarized in Table 1.

The mean age was 65 years, mean PSA was 8.86 ng/mL, mean free to total PSA ratio was 14.26%, and mean prostate volume was 30.97 mL. Clinical T1c patients were the most common, and 127 cases (80%) and 5 cases (3%) of clinical T3a were included. The biopsy Gleason score was 6 in 48 cases (31%), 7 in 86 cases (54%), and 8 or more in 24 cases (15%). The RP Gleason score was 6 in 16 cases (10%), 7 in 120 cases (76%), and 8 or more in 22 cases (14%). RP was performed by open laparotomy in 50 cases and was laparoscopic in 108 cases.

Table 2 lists the location of cancer in all 158 cases. In the prostatectomy specimens, cancer was found more frequently in the apex 85% than the middle 77%, base 22%, or TZ 22% of all RP patients. This trend was the same in the anterior area (apex, middle, base, and TZ were 77%, 53%, 13%, and 16%, resp.). The “apex anterior” location was the most frequent among the 158 patients studied (122, 77%).

Table 3 presents concordance rates for prostatectomy specimens and needle biopsy results, as calculated for each of the biopsy core locations (584 sections from 73 patients diagnosed at our institution). For all sections evaluated, 161 (51%) were true positives, 153 (49%) were false negatives, 45 (17%) were false positives, and 225 (83%) were true negatives. The sectional false negative rate was 45% in apex, 45% in middle, 48% in base, and 49% in TZ specimens. The true positive rate was worst (38%) in specimens from the base.

“Apex” was the most frequent cancer area identified, and the false negative rate was 45%. The apex is one of the most important locations of prostate cancer in Japanese RP patients.  Table 4 lists univariate and multivariate analyses of factors used to detect apex cancer in 65 true positive and 53 false negative cancers: 40 (75%) of the 53 false negative cancers were significant cancers. “Insignificant” cancer was defined as Gleason score 3 + 3 or less, organ-confined cancer and tumor volume of 0.5 mL or less. In univariate analysis, significant differences were observed in the apex for the free to total PSA ratio, positive core number, pathological stage, apex tumor volume, and total tumor volume (*P* = 0.0240, *P* = 0.0002, *P* = 0.010, *P* ≤ 0.0001, and *P* = 0.016, resp.), but not for age, body mass index, PSA level, prostate volume, clinical stage, biopsy Gleason score, or RP Gleason score. In multivariate analysis, apex tumor volume was the only independent factor of all the clinicopathological characteristics analysed (*P* = 0.0002). No factors readily available preoperatively correlated with detection of apex cancer by transrectal biopsy specimens.

## 4. Discussion

In the present study, no readily available preoperative factor was found to correlate with detection of apex prostate cancer by transrectal biopsy. In addition, there were too many significant cancers identified falsely as negative using this transrectal biopsy procedure. 

Prostate cancers occurred most frequently (85%) in the apex, confirming a previous report made in 2002 [7]. The working hypothesis leading to this study was that, because of widespread screening for PCA and improved imaging technology, the RP patient population might have changed. However, the trend seen was not different from that observed 10 years ago. The study population was small, a fact that might influence the results. On the other hand, the location of prostate cancer in Japanese men differed from that seen in the United States [5, 6]. These findings suggest there may be some racial differences regarding PCA localization. 

A previous report of 66 patients with no history or clinical evidence of PCA demonstrated that 38% had tumors with a mean volume of 0.11 mL, and these were located exclusively in the apex [11]. However, when peripheral zone cancers greater than 4 mL in volume were found, they appeared to be directed toward the base [12]. Thus, one hypothesis is that most PCAs found incidentally, especially peripheral zone cancers, arise in the apex and spread toward the base. Takashima et al. indicated that clinically favorable cancers are located preferentially in the apex. It follows that a positive biopsy core from the apex may more likely be a clinically indolent cancer than a positive core from the middle or base areas [7].

Prostate biopsy procedures are becoming less random and more systematic, but cancer is still being missed. The current standard of care practice for an initial biopsy involves taking 10 to 14 cores, a procedure that detects PCA up to 40.3% of the time [13–17]. Previously, we showed that the cancer detection rate for the 8, and 14-core groups was 14.5% (16 of 110 patients) and 24.5% (23 of 94 patients), respectively [18]. Findings of the current study demonstrate that, despite use of appropriate techniques, transrectal prostate biopsy alone does not provide a high tumor detection rate; 49% of all areas biopsied were false negatives, as were 45–49% of each area analyzed. Reasons for false negative occurrence may differ among areas biopsied. In the apex, the occupied volume is small and the angle attainable by the transrectal approach might be limited, which is the reverse of the situation in the prostate base. 

The “apex” is the most frequent location of PCA and there is a high false negative rate from transrectal biopsy. Orikasa investigated the utility of directing biopsies to the apical anterior peripheral zone (AAPZ). From initial 12-core biopsies, 50.8% (128/252) of cancers were detected in AAPZ cores. Although an increase of overall cancer detection in the apical anterior biopsies was modest, 5.2% of cancers were detected only from AAPZ cores in initial biopsy material. In repeat biopsy specimens, 36.0% of the cancers were found exclusively in the AAPZ and the detection rate from this zone was significantly higher than that in initial biopsy cores. It is important to note that the AAPZ biopsy strategy had greater utility in men with normal DRE, and particularly in men with a prior negative biopsy [19]. Jonathan directed the biopsy more peripherally, approximately 3 mm below the capsule, and demonstrated that this procedure makes inadvertent sampling of the transition zone less likely. As a result, the anterior apex was found to be the most frequent site of unique cancer detection. Including cores obtained in this way increased the overall cancer detection rate to 40.9% [20]. The apex is the most common positive resection margin (PRM) site following RP, with a frequency of up to 55.8% [21–23]. 

In the present study, the factors predicting apex cancer detection in transrectal biopsy specimens were analyzed for sensitivity and specificity. Apex tumor volume was the only independent factor found. No preoperative factors were found to be predictive. It has long been known that PCA tumor volume correlates well with common adverse features such as high Gleason score, extraprostatic extension, seminal vesicle invasion, and clinical outcome [24, 25]. However, in the current study, a positive biopsy from the apex was not predicted by PSA level, Gleason score, stage, or total tumor volume, but only by the apex tumor volume. We had developed a nomogram predicting the probability of a positive initial prostate biopsy in Japanese patients having serum PSA levels less than 10 ng/mL. Age and other possible independent predictors of a positive biopsy, such as elevated PSA, decreased free to total PSA ratio, small prostate volume, and abnormal digital rectal examination findings, were used previously to develop a predictive nomogram [26]. These factors are commonly used for predicting the probability of a positive initial prostate biopsy. In actuality, this study demonstrates the limitations of detecting apex tumors using only transrectal biopsy material. Improved imaging technology or carrying out additional transrectal biopsies or addition of transperineal biopsies is needed to improve apex biopsy accuracy.

Limitations of this study include its retrospective nature and relatively small number of patients. Results could have been biased by patient selection for RP and biopsy. It is difficult to definitively localize PCA and identify an optimal biopsy strategy or even the optimal indication for biopsy. Nevertheless, even with these limitations, the current results suggest that it is difficult to predict apex cancer preoperatively using methods currently available.

## 5. Conclusions

In Japanese patients, the apex was the most frequent location of prostate cancer and a high false negative rate was found for transrectal biopsy. It is difficult to predict apex cancer preoperatively using methods currently available.

## Figures and Tables

**Table 1:** Patients' characteristics.

Characteristic	Study population (*n* = 158)
Age, mean ± SD years	65.26 ± 5.11
PSA, mean ± SD ng/mL	8.86 ± 5.09
PSA F/T, mean ± SD %	14.26 ± 7.66
Clinical T stage	
T1c	127 cases
T2a–c	26 cases
T3a	5 cases
Biopsy Gleason score	
6	48 cases
7	86 cases
*⩾*8	24 cases
Prostate volume, mean ± SD mL	30.97 ± 15.20
Operation	
ORP	50 cases
LRP	108 cases
Pathologic T stage	
T2a–c	98 cases
T3ab	59 cases
T4	1 case
RP Gleason score	
6	16 cases
7	120 cases
*⩾*8	22 cases

PSA: prostate specific antigen, F/T: free-to-total PSA ratio, RP: radical prostatectomy, ORP: open radical prostatectomy, LRP: laparoscopic radical prostatectomy.

**Table 2:** Presence of cancer in each location of all 158 radical prostatectomy patients.

	Anterior	Posterior	Ant or post
Apex	122 (77%)	78 (49%)	134 (85%)
Middle	83 (53%)	81 (51%)	122 (77%)
Base	21 (13%)	22 (13%)	35 (22%)
TZ	25 (16%)	18 (11%)	35 (22%)
Any section	135 (85%)	120 (76%)	—

Ant: anterior, post: posterior.

**Table 3:** Concordance rate of prostatectomy specimen and needle biopsy. *n* = 584. 73 (patient) × 8 (section).

Location of RP specimen	Biopsy tumor (+)	Biopsy tumor (−)	Total
Apex			
Tumor (+)	65 (55%)	53 (45%)	118
Tumor (−)	2 (7%)	26 (93%)	28
Middle			
Tumor (+)	54 (55%)	44 (45%)	98
Tumor (−)	6 (13%)	42 (87%)	48
Base			
Tumor (+)	23 (38%)	38 (48%)	61
Tumor (−)	21 (25%)	64 (75%)	85
TZ			
Tumor (+)	19 (51%)	18 (49%)	37
Tumor (−)	16 (15%)	93 (85%)	109
Any section			
Tumor (+)	161 (51%)	153 (49%)	314
Tumor (−)	45 (17%)	225 (83%)	270

**Table 4:** Univariate and multivariate analysis of factors for detecting apex cancer among clinicopathological factors.

	True positive	False negative	Univariate analysis	Multivariate analysis	
	*n* = 65	*n* = 53	*P* value	95% CI
Age, mean years	65.2	65.3	0.898		
BMI, mean kg/m^2^	22.4	22.6	0.677		
PSA, mean ng/mL	9.69	8.10	0.113		
F/T ratio, mean %	12.8	16.4	0.024*	0.953–1.100	0.737
Prostate volume, mean mL	32.1	32.5	0.890		
Clinical T stage					
T1c	50	40			
T2a–c	12	12	0.954		
*⩾*T3a	3	1			
Biopsy Gleason score					
6	20	24			
7 8	41	27	0.197		
9 10	4	2			
Positive core number, mean	3.66	2.43	0.0002*	0.574–1.133	0.215
pathological T stage					
T2a–c	33	39	0.010	0.234–2.632	0.696
*⩾*T3a	32	14			
RP apex Gleason score					
6	13	13			
7 8	46	35	0.431		
9 10	6	5			
Apex tumor volume, mean mL	0.802	0.193	<0.0001*	0.004–0.192	0.0002*
Total tumor volume, mean mL	2.76	1.76	0.016*	0.572–1.001	0.051

True positive: RP specimen positive and biopsy positive, false negative: RP specimen positive and biopsy negative, BMI: body mass index, PSA: prostate specific antigen, F/T: free total PSA ratio, RP: radical prostatectomy, *statistically significant.

## Abbreviations

PCA: Prostate cancer
TZ: Transitional zone
RP: Radical prostatectomy
PSA: Prostate specific antigen
DRE: Digital rectal examination
TRUS: Transrectal ultrasound
AAPZ: Apical anterior peripheral zone
PRM: Positive resection margin.
